# Effects of different psychological interventions on quality of life and remission rate in patients with acute leukemia receiving chemotherapy: A randomized controlled trial

**DOI:** 10.3389/fpsyg.2023.1045031

**Published:** 2023-02-16

**Authors:** Fang Peng, Huiyuan Li, Jingping Zhang, Xiaoyun Li, Haimiao Zhang, Yifei Li

**Affiliations:** ^1^Changsha Social Work College, Changsha, China; ^2^The Nethersole School of Nursing, Faculty of Medicine, The Chinese University of Hong Kong, Shatin, Hong Kong SAR, China; ^3^Xiangya School of Nursing, Central South University, Changsha, China; ^4^Second Xiangya Hospital of Central South University, Changsha, China; ^5^The School of Nursing and Rehabilitation, Xi’an Medical University, Xi’an, China

**Keywords:** acute leukemia, cognitive intervention, progressive muscle relaxation, quality of life, remission

## Abstract

**Aims:**

This study aimed to examine and compare different psychological intervention effects on the quality of life (QoL) and remission rates of patients with acute leukemia receiving chemotherapy.

**Methods:**

A total of 180 participants were randomly divided into a cognitive intervention group, a progressive muscle relaxation (PMR) group, a cognitive intervention plus PMR group, and a usual care control group. QoL via the Chinese version of the European Organization for Research and Treatment of Cancer Quality of Life Questionnaire Core-30 and remission rate were assessed at baseline and immediate post-intervention. A Generalized Linear Mixed Model was used for statistical analysis. Cost-effectiveness analysis with the value of the Incremental Cost-effectiveness Ratio was conducted to realize the economic evaluation of psychological interventions.

**Results:**

The total score of QoL and its most dimensions were significantly improved for participants in the intervention groups compared with the control group. The cognitive intervention plus PMR intervention was the most effective concerning QoL with cost-effectiveness. No significant improvements were indicated in participants’ remission rates among the groups.

**Conclusion:**

The cognitive intervention plus PMR intervention is the most effective in improving QoL with cost-effectiveness among patients with acute leukemia receiving chemotherapy. More rigorous randomized controlled trials with multiple follow-up points are suggested to clarify the psychological interventions on remission rates in this population.

## Introduction

Acute leukemia (AL) is a malignant clonal disease originating from hematopoietic stem cells, the most common hematologic cancer worldwide (Khan et al., 2018). As an aggressive disease develops rapidly, patients often require immediate hospitalization to initiate intensive chemotherapy (Byrant et al., 2018). Patients with AL treated with chemotherapy tend to face many challenges. Not only the AL disease itself but also AL-related chemotherapy impair physical function (Oztürkmen et al., 2010; Musarezaie et al., 2014; Miladinia et al., 2018), resulting in fatigue, diarrhea, nausea, and vomiting (Burns et al., 2008). Mental health is also adversely affected in patients receiving chemotherapy for leukemia. Many patients experience high-level psychological distress due to the protracted time required for chemotherapy, high treatment cost, and less than optimal therapeutic effects (Albrecht, 2014; Wang et al., 2021). Decreased physical functions also impede their responsibility fulfillment in their families and societies and cause dramatic changes in lifestyles (Jie et al., 2020), contributing negatively to a downward spiral of physical deconditioning that affects physical and psychological functions, finally causing a poor quality of life (QoL) (Byrant et al., 2018; Nørskov et al., 2019).

The World Health Organization (WHO) has emphasized that successful leukemia treatments must improve patients’ survival rate and QoL (Khan et al., 2018). QoL is a multidimensional concept and subjective experience determined by patients’ physical, psychological, and social health (Chabowski et al., 2018; Wang et al., 2020). Studies have shown that the QoL for patients with AL needs to be improved (Bryant et al., 2015). However, existing studies have mainly focused on the effects of anti-leukemic treatments (i.e., chemotherapy and stem cell transplantation) in AL patients (Messerer et al., 2008; Grulke et al., 2012), failing to pay sufficient attention to different aspects of the well-being of AL patients. Meanwhile, remission rates directly affect survival and are the ultimate objective of chemotherapy among AL patients (Blackburn et al., 2019). Achieving high-quality complete remission in the short term can help patients kill more leukemia cells before developing secondary resistance and prolong the patient survival rates (Ravandi, 2014). However, remission rates (Ravandi, 2014; Yoon et al., 2017) are often used as assessment indicators for pharmacological studies, with little consideration of remission as a dependent variable in nonpharmacological interventions. Therefore, identifying effective nonpharmacological interventions that enhance QoL and promote remission rates for patients with AL receiving chemotherapy is particularly important.

Among various nonpharmacological interventions, psychological intervention is one of the most commonly used means to improve cancer patients’ QoL and relieve their diverse distress aspects, especially cognitive-based therapy and progressive muscle relaxation (PMR). Cognitive therapy (Schilder, 1953) is a form of structured, short-term, and present-oriented psychotherapy to alter harmful cognitions by changing individuals’ thoughts, beliefs, and behaviors. Previous studies suggested its promising effects in reducing the side effects of cancer-related treatments and improving cognitive and emotional functions and QoL in cancer patients (Munir et al., 2011; Bail et al., 2020). Despite the impacts of cognitive difficulties on cancer patients, evidence claiming that cognitive therapy improves QoL in patients with AL receiving chemotherapy is scant.

PMR is a complementary and alternative medicine intervention (Park, 2013) that includes repetitive cycles of tensing and relaxing major muscle groups combined with breathing exercises (Jacobson, 1938). A recent systematic review has demonstrated that PMR benefits patients undergoing chemotherapy, not only diminishing stress but also alleviating anxiety and side effects caused by chemotherapy. However, the effects of PMR on improving QoL remain unclear in patients with AL receiving chemotherapy.

Psychological interventions have been evaluated in patients with solid tumors. Still, as a kind of hematologic malignancy, patients with AL receiving chemotherapy are rarely studied as a single population, and few psychological interventions (Gray et al., 2021) have been conducted in these patients. Compared with patients with solid tumors, many patients with hematologic malignancies require intensive treatments. At the expense of substantial toxicities and impairments to QoL, it causes a double burden on patients’ physical and mental health (El-Jawahri et al., 2020). Literature reports that psychological factors (i.e., depression and anxiety) directly affect disease occurrences and outcomes (Messerer et al., 2008), impacting AL patients’ remission (Albrecht, 2014; Zhang et al., 2021). Cognitive intervention has also indicated its promising effects in improving psychological well-being in cancer patients (Compen et al., 2018). However, the effects of cognitive intervention effects on improving remission rates by correcting cognitions to improve psychological outcomes remain unclear. In addition, although cognitive intervention and PMR have been widely used in cancer patients, cognitive intervention plus PMR based on changing patient cognition has rarely been carried out. A single intervention program may no longer meet patients’ physical and mental care demands during treatments. Developing an intervention that combines cognitive intervention and PMR to improve QoL and remission in patients with AL receiving chemotherapy is necessary.

This study aimed to examine and compare the effects of three psychological interventions on the QoL and remission rates of patients with AL receiving chemotherapy. The results will be beneficial in identifying the best intervention for improving survival outcomes for this population.

## Materials and methods

### Research design and participants

**Figure 1:**
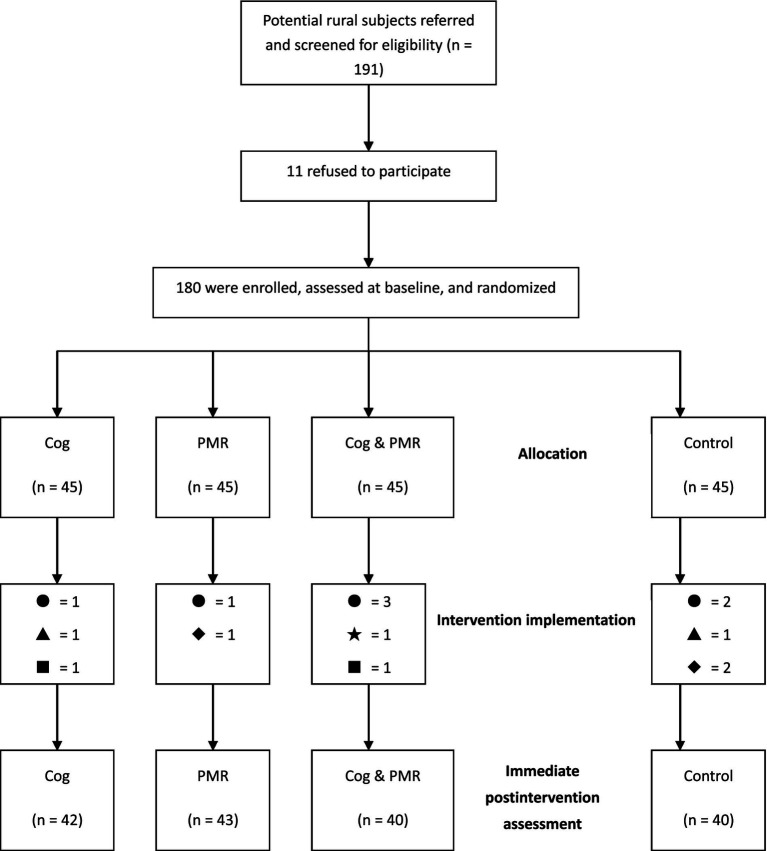
Study procedure. Cog, cognitive intervention; PMR, progressive muscle relaxation.

A randomized, assessor-blind, controlled trial design (RCT) was adopted. The study was conducted for 4 months, from July 2009 to November 2009, in three university-affiliated hospitals in Central China, comprising a baseline survey, an intervention implementation and conclusion, and an immediate postintervention assessment (Figure 1). This RCT adhered to the CONSORT Statement.

The inclusion criteria were as follows: (1) diagnosed with AL based on bone marrow morphology, histochemistry, and immunophenotype; (2) aged ≥18 years; (3) had just begun chemotherapy; (4) Karnofsky Performance Status (KPS) scale score ≥ 60; and (5) were able to provide written informed consent.

Participants who (1) were receiving concurrent radiation therapy or bone marrow transplantation; (2) had other severe physical illnesses; (3) had difficulty with self-expression due to psychological or cognitive issues; or (4) refused to participate or complete questionnaires were excluded from the study.

The sample size estimate per group (*n* = 36) was calculated using G*Power based on the effect size *d* = 0.73, two-trailed *α* = 0.05, and 1-*β* = 0.86 (Amir and Ramati, 2002). As a 20% attrition rate was anticipated, the total sample size was 180, with 45 patients per group.

### Randomization

Clinical nurses reviewed medical charts and identified eligible participants according to eligibility criteria. The researchers then approached and explained the study aim and procedure. Those who agreed to participate gave written informed consent and completed a baseline assessment. The randomization procedure was then performed by a research assistant who was not involved in the recruitment, enrollment, and treatment process. Participants were randomized at a 1:1:1:1 ratio to the intervention groups or control group using an independent web-based randomization system. The research assistant, who performed randomization, informed the participants about the allocation using an opaque and sealed envelope.

### Procedure

A total of 180 patients were recruited and randomly divided into four groups. Each group had 45 patients based on a random number table. The three intervention groups received cognitive intervention (G1), PMR (G2), and cognitive intervention plus PMR intervention (G3) apart from routine nursing care. The interventions in the three groups were provided by the principal investigator (FP), who has a master’s degree in nursing at the time of intervention implementation, with extensive experiences in hematologic cancer care and psychological support. The interventionist received the cognitive intervention and PMR training from experienced therapists and experts for 1 year. Before the intervention procedure, the interventionist adopted the standardized intervention manual and practiced with research assistants until fidelity was achieved. During the intervention process, the interventionist received weekly supervision (JZ) to ensure adherence to the study protocol, and self-reflection diaries were documented. The control group (G4) received only routine nursing care, including hospital education and general nursing procedures. All participants completed questionnaires at baseline (T1: the day after admission) and immediately after the intervention (T2). Each group’s intervention was conducted in an independent department office, separated from patients’ activity area. Participants in each group were encouraged not to discuss the content of the intervention they received with others to avoid contamination.

### Cognitive intervention

Interventions were carried out as group activities, with three to five participants in each group. The cognitive intervention comprised three weekly sessions (50–60 min/session). The interventionist directed each activity, guided group discussions, recorded each participant’s main idea, and summarized every activity. Each was encouraged to participate in three sessions and completed written assignments after each session.

The first session sought to confirm the negative factors that affect their emotion management. The specific approach was establishing good relationships within patient groups and helping participants recognize depression and emotional disturbances after hospitalization. Furthermore, patients were taught standard methods of psychological self-adjustment, emotion management, and basic knowledge of AL chemotherapeutic treatment.

The second session focused on the recognition of and response to social support, with an emphasis on positive thinking and the use of social support resources. Participants were encouraged to change negative and irrational perceptions and form positive thinking, such as focusing on the good aspects of any given situation. They were also suggested to cultivate active coping styles. The interventionist explained how to effectively use social support resources by combining the content of the expression of each participant.

The third session comprised education, encouragement, and future perspective. Each participant shared a successful experience with the group to build confidence and defeat the illness. The interventionist guided them to appreciate the life meaning and helped them determine their expectations and objectives for the future. Last, the interventionist helped the participants review and examine their understanding of the contents of each cognitive group session.

#### Progressive muscle relaxation (PMR)

PMR was used as the muscle relaxation intervention without interfering with cancer treatment. An experienced interventionist supervised the participants in 20–30-min PMR twice or thrice daily within 3 weeks.

The relaxation movement was conducted in consecutive steps (McCallie et al., 2006). (1) The interventionist provided a quiet and comfortable environment for relaxation with temperature maintained at 22°C–28°C. Participants were told to lie down evenly and rest for 10 min with their minds focused and without distracting thoughts. (2) Testing and recording the skin electromyography value before relaxation. (3) The interventionist guided the participants to relax with music from an MP3 player (produced by Chinese Medical Multimedia Press) and carry out PMR with each body part. The essential action points of PMR included: tense muscles, paying attention to this feeling of tension, maintaining this tension for 10 s, then relaxing for 5–10 s, and experiencing the sense of the muscles while relaxing. (4) Re-testing and recording skin electromyography after relaxing the muscles.

Signs of successful relaxation included: (1) absence of facial expressions, (2) every part of the muscle was slack, (3) tension in the limbs and neck was relieved, (4) breathing slowed, (5) the feet would abduct when the patient reclined on their back.

Skin electromyography was recorded by a computer biofeedback instrument for monitoring (JD-2A), which is a kind of precision electronic equipment for psychosomatic disease prevention and treatment using the feedback signal of electromyography. The instrument uses sensors to detect the electrical signals of participants’ muscles. After amplifying and filtering the signs, the analog quantity is converted into a digital portion through A/D conversion. The muscle activity is expressed by numbers, cursors, and sounds and fed back to trainees. Thus, participants can consciously regulate and control their physiological functions and reshape their emotions, visceral activities, and physical behaviors to achieve the purposes of curing diseases and recovering. Therefore, the utilization of recording skin electromyography *via* JD-2A can realize: (1) Monitoring (Patients’ mastery of relaxation techniques may directly affect intervention effects, so the biofeedback signal of skin electromyography can help observe whether participants achieve true relaxation after the intervention); and (2) Feedback (Through the feedback function of the instrument, patients can get timely feedback while performing relaxation, which allows them to adjust the technique dynamically and better master the relaxation technique).

#### Cognitive intervention plus PMR

The intervention combined the abovementioned cognitive intervention and PMR within 3 weeks. The interventionist conducted PMR first per day. After participants completed this section 2–3 times, each session of the cognitive intervention was conducted in order. Each participant in this group was encouraged to finish the whole intervention program, including the cognitive intervention and PMR part.

### Measurements

#### Sociodemographic questionnaire

The questionnaire included basic demographic information (e.g., sex, age, profession, education level, marital status, and economic condition) and relevant clinical characteristics (e.g., diagnosis, chemotherapy session, number of hospitalizations, satisfaction with medical and nursing care).

#### European organization for research and treatment of cancer quality of life questionnaire core-30

EORTC-QLQ-C30 (Grulke et al., 2012) is an established QoL questionnaire for cancer patients, including five functional sub-questionnaires (physical, role, emotional, cognitive, and social functions); three symptom sub-questionnaires (weariness, nausea and vomiting, and pain); one sub-questionnaire on general health condition; and six single-factor entries (polypnea, insomnia, inappetence, constipation, diarrhea, and economic difficulties). The greater the score for general health/QoL, the better the participant’s condition; the greater the score for each sub-questionnaire, the poorer the participant’s condition. The Chinese version showed acceptable reliability and good construct validity in measuring QoL among Chinese cancer patients (Wan et al., 2008). Cronbach’s α ranged from 0.86 to 0.91 for the total scale and subscales in the present study.

### Remission

Physicians judged clinical remission on the basis of the Standard of Diagnosis and Therapeutic Effect of Hematopathy (Zhang and Shen, 2007), which categorizes remissions as complete remission (CR), partial remission (PR), and non-remission (NR). Indices for judgment often include clinical manifestations, physical signs, hemograms, and bone marrow characteristics. For AL, CR refers to the absence of leukemic cell infiltration, clinical signs and symptoms, with the patient’s life being normal or near normal, Hb ≥ 100 g/l (male) or ≥ 90 g/l (female and children), platelets ≥100 × 109/L, neutrophils ≥100 × 109/L, absence of leukemic cells in the peripheral blood, and changes in the bone marrow.

PR refers to myeloid progenitor cell types 1 and 2 (monoblast + promonocyte or lymphoblast + prolymphocyte) > 5% but ≤20%, failing to meet the CR standard for clinical symptoms or hemograms.

NR (recurrence) refers to one of the following three conditions after complete remission: (1) Myeloid progenitor cell types 1 and 2 (monoblast + promonocyte or lymphoblast + prolymphocyte) > 5% but ≤20%, with a myelogram that fails to meet the CR standard after a course of an effective anti-leukemia treatment; (2) Myeloid progenitor cell types 1 and 2 (monoblast + promonocyte or lymphoblast + prolymphocyte) > 20%; and (3) extramedullary leukemia cell infiltration.

### Data analysis

Data collection and sorting were accomplished promptly. Baseline assessment (T1) before randomization and immediate postintervention assessment (T2) were conducted by two independent and trained assessors who were blinded to the study. Database construction, data input, and statistical analysis were all carried out under statistical expert supervision.

SPSS 22.0 was used to establish the database. After inputting data, the researcher used SPSS to sort data and check for logic errors. The GLIMMIX macro process of SAS 9.4 was employed to fit a generalized linear mixed model (GLMM) of measurement data and repeated measurement data. The GLMM was used for parameter estimation and hypothesis testing (Dean and Nielsen, 2007). Descriptive analysis was performed for general data. The Chi-square test was performed to compare data among the four groups. The GLMM was conducted to analyze interaction effects on QoL and remission among groups across time.

In addition, the economic evaluation of the three psychological interventions encompassed a cost-effectiveness analysis (CEA) (Sanders et al., 2019). Incremental Cost-effectiveness Ratio (ICER), an economic value of an intervention compared with an alternative way, was calculated by the difference in cost between target intervention and usual care, divided by the difference in their effect (Chen et al., 2020). It represents the average incremental cost associated with one additional unit of the effect measure. ICERs are most valuable when the new intervention is costly but generates improved health effects. It can help contain health care costs while minimizing adverse health consequences (Dakin et al., 2015). ICERs reported by economic evaluations are compared with a predetermined threshold to decide whether choosing the new intervention is an efficient resource use.

### Ethical consideration

The study was conducted following the Helsinki Declaration of the World Medical Association Assembly. The experimental procedure was approved by the Clinical Research Ethics Review Committee of Central South University. Research significance, anonymity principles, collected data confidentiality, and the right to leave the study at any time without any penalty were explained to participants. All individuals participated voluntarily and signed the informed consent form before randomization.

## Results

### Demographics and clinical characteristics

A total of 180 participants were recruited for the study, with 45 participants in each group. At the end of the study, a total of 165 participants completed the whole intervention with a completion rate of 91.7%. The number of valid participants who completed the study in each group was 42, 43, 40, and 40, respectively. Reasons for lack of completion were failure to keep in contact (*n* = 7), quitting the study (*n* = 3), illness exacerbation (*n* = 2), being discharged (*n* = 2), or therapy changed to bone marrow transplantation (*n* = 1).

The mean age was 34.52 ± 13.27 (14–66) years. Most participants were males (*n* = 93, 56%), married (*n* = 102, 62%), and completed high school or polytechnic school (*n* = 66, 40%). No between-group differences in demographics or clinical characteristics were observed (*p* > 0.05), except ethnic group and years of disease course (*p* < 0.05; Table 1).

**Table 1:** Sample demographics and clinical characteristics.

Item	Classification	Cognitive group *n* = 42		PMR group *n* = 43		Cognitive plus PMR group *n* = 40		Control group *n* = 40		*χ* ^2^	*p^a^*
		*n*	%	*n*	%	*n*	%	*n*	%		
Sex	Male	20	48	21	49	24	60	28	70	5.54	0.137
	Female	22	52	22	51	16	40	12	30		
Age	≤35	18	43	28	65	18	45	20	50	5.14	0.162
	35	24	57	15	35	22	55	20	50		
Ethnic group	Han	40	95	33	77	37	93	38	95	21.71	0.007^**^
	Ethnic minor	2	5	10	23	3	8	2	5		
Religion	No	40	2	42	98	36	90	37	93	2.41	0.426
	Yes	95	5	1	2	4	10	3	7		
Residence	Country	19	45	24	56	24	60	23	58	2.12	0.549
	Town	23	55	19	44	16	40	17	42		
Marital status	Married	28	67	24	56	26	65	24	60	8.52	0.708
	Single/divorced	14	33	19	44	14	35	16	40		
Junior middle school and below	Junior middle school and below	22	52	14	33	12	30	15	38	3.51	0.319
	High school or polytechnic	11	26	16	37	21	53	18	45		
	College and above	9	21	13	30	7	18	7	18		
Occupation	Workman	7	17	4	9	3	7	6	15	25.8	0.211
	Farmer	14	33	13	30	9	23	10	25		
	Official	2	5	2	5	6	15	0	0		
	Teacher	5	12	4	9	4	10	3	7		
	Student	8	19	9	21	8	20	9	23		
	Other	3	7	1	2	1	2	0	0		
	Unemployed	3	7	10	24	9	23	12	30		
Economic condition	Very poor	11	26	14	33	8	20	10	25	5.31	0.151
	Poor	4	10	11	26	4	10	5	13		
	Ordinary	25	60	14	33	22	55	23	58		
	Rich	2	5	4	9	6	15	2	5		
Admission diagnosis	AML	21	50	29	67	26	65	24	60	3.15	0.369
	ALL	21	50	24	23	14	35	16	40		
Disease course year	≤1 year	35	81	26	60	36	90	34	85	9.80	0.020
	>1 year	7	19	17	40	4	10	6	15		
Initial treatment	Yes	13	31	8	19	8	20	9	23	2.18	0.537
	No	29	69	35	81	32	80	31	78		
Phase of chemotherapy	Induction therapy	21	50	13	30	15	38	14	35	9.32	0.156
	Consolidation therapy	13	31	11	26	15	38	16	40		
	Maintenance therapy	8	19	19	44	10	25	10	25		
Hospitalization frequency	1–5	28	67	27	63	28	70	30	75	4.85	0.563
	6–10	12	29	13	30	11	28	6	15		
	11–15	2	5	3	7	1	3	4	10		
Other diseases	Associated	6	14	9	21	9	23	4	10	2.94	0.401
	Unassociated	36	86	34	79	31	78	36	90		
KPS score	90	10	24	7	16	9	23	7	18	0.81	0.847
	80	11	26	14	33	16	40	15	38		
	70	11	26	14	33	10	25	11	28		
	60	10	24	8	19	5	13	7	18		

^a^Value of *p* for controls vs. patients based on the chi-square test for categorical data. PMR, progressive muscle relaxation.

### Changes in QoL

The raw score for general health conditions was 4.11 ± 1.21. After conversion, all standard scores for function dimensions, symptom dimensions, and six single-factor dimensions decreased after interventions, with scores for general health improved (Table 2).

**Table 2:** Quality of life measurement scale scores before and after interventions [ $\bar{x}$ ± standard deviation (SD)].

Dimension	PMR (*n* = 43)		Cognitive intervention (*n* = 42)		Cognitive intervention plus PMR (*n* = 40)		Control group (*n* = 40)	
	Before	After	Before	After	Before	After	Before	After
Functional dimensions								
Physical function	2.23 ± 0.10	1.60 ± 0.35	2.20 ± 0.11	1.90 ± 0.45	2.17 ± 0.11	1.60 ± 0.36	2.17 ± 0.11	1.90 ± 0.56
Role function	2.78 ± 0.13	1.90 ± 0.63	2.40 ± 0.13	1.80 ± 0.62	2.66 ± 0.14	1.50 ± 0.40	2.55 ± 0.14	2.66 ± 0.12
Emotional function	1.67 ± 0.07	1.60 ± 0.46	1.70 ± 0.07	1.70 ± 0.38	1.56 ± 0.08	1.50 ± 0.42	2.25 ± 0.08	2.25 ± 0.62
Cognitive function	1.67 ± 0.09	1.40 ± 0.44	1.50 ± 0.09	1.20 ± 0.35	1.89 ± 0.09	1.40 ± 0.44	1.78 ± 0.09	1.95 ± 0.63
Social function	2.70 ± 0.12	2.40 ± 0.76	2.50 ± 0.12	2.00 ± 0.75	2.55 ± 0.13	1.90 ± 0.62	2.43 ± 0.13	2.25 ± 0.77
Symptom dimensions								
Weariness	2.33 ± 0.09	1.80 ± 0.62	2.40 ± 0.09	1.70 ± 0.52	1.93 ± 0.09	1.50 ± 0.44	2.40 ± 0.09	2.11 ± 0.66
Nausea and vomiting	2.23 ± 0.12	1.50 ± 0.48	1.80 ± 0.12	1.40 ± 0.41	2.05 ± 0.13	1.40 ± 0.44	2.05 ± 0.13	1.78 ± 0.65
Pain	1.97 ± 0.10	1.50 ± 0.08	2.10 ± 0.10	1.76 ± 0.09	2.19 ± 0.11	1.65 ± 0.09	2.20 ± 0.11	1.99 ± 0.09
Single dimensions								
Polypnea	1.61 ± 0.10	1.30 ± 0.47	1.80 ± 0.11	1.50 ± 0.59	1.68 ± 0.11	1.20 ± 0.42	1.85 ± 0.11	1.63 ± 0.59
Insomnia	2.00 ± 0.14	1.40 ± 0.63	2.10 ± 0.14	1.70 ± 0.84	2.23 ± 0.14	1.70 ± 0.69	2.18 ± 0.14	2.13 ± 0.88
Inappetence	2.49 ± 0.14	1.50 ± 0.50	2.30 ± 0.14	1.70 ± 0.66	2.05 ± 0.14	1.60 ± 0.66	2.13 ± 0.14	2.05 ± 0.82
Constipation	1.72 ± 0.12	1.40 ± 0.50	1.50 ± 0.12	1.40 ± 0.63	1.83 ± 0.12	1.10 ± 0.39	1.73 ± 0.12	1.63 ± 0.71
Diarrhea	1.77 ± 0.11	1.56 ± 0.09	1.4 ± 0.11	1.24 ± 0.09	1.60 ± 0.11	1.43 ± 0.09	1.70 ± 0.11	1.50 ± 0.09
Economic difficulty	3.14 ± 0.14	3.40 ± 0.62	2.90 ± 0.15	3.10 ± 0.70	2.85 ± 0.15	3.00 ± 0.75	3.08 ± 0.15	3.05 ± 0.64
General health	3.92 ± 0.19	5.30 ± 0.84	4.10 ± 0.19	5.30 ± 0.71	4.13 ± 0.19	5.50 ± 0.74	4.26 ± 0.19	4.93 ± 0.69

PMR, progressive muscle relaxation.

### Effects of the psychological interventions on QoL

In the GLMM model, the total score of QoL and score for each dimension were set as response variables. The fixed effects involved the intervention group, time, ethnic group, disease course, and intervention–time interactions; meanwhile, the random effects involved individual factors. The model was used as a covariate to account for the confounding effects of ethnic group and disease course. The results demonstrated a main intervention effect on the total QoL score (*p* < 0.05). The main time effect was insignificant (*p* > 0.05). An interaction was found between intervention and time (*p* < 0.05). No significant differences were observed for ethnic group or disease course (*p* > 0.05; Table 3). Regarding the total score, in the GLMM model (Table 4), the estimated values for groups 1, 2, and 3 were − 0.10, −0.11, and − 0.15, respectively. These results demonstrated the efficacy of the experimental groups compared with that of the control group. The advantage efficacy of the experimental groups was 1.02 times greater than the control group. The estimated value for the time group was 0.02, demonstrating that the QoL after interventions significantly improved (Table 4).

**Table 3:** Type III fixed effects of quality of life (*n* = 165).

Effect		Group		Time		Ethnic group		Disease course		Group*time	
		Num DF ^a^3	Den DF ^b^159	Num DF 1	Den DF 160	Num DF 1	Den DF 159	Num DF 1	Den DF 161	Num DF 3	Den DF 160
Total score	*F*	4.71		94.51		1.06		1.57		6.74	
	*p*	<0.01^**^		<0.01^**^		0.30		0.21		<0.01^**^	
Physical function	*F*	1.41		128.08		4.47		4.84		4.55	
	*p*	0.24		<0.01^**^		0.04^*^		0.03		<0.01^**^	
Role function	*F*	3.42		88.09		2.44		1.68		16.46	
	*p*	0.02^*^		<0.01^**^		0.12		0.20		<0.01^**^	
Emotional function	*F*	11.75		0.00		2.08		46.60		0.00	
	*p*	<0.01^**^		1.00		0.15		<0.01^**^		1.00	
Cognitive function	*F*	6.21		16.87		0.42		0.36		9.07	
	*p*	<0.01^**^		<0.01^**^		0.52		0.55		<0.01^**^	
Social function	*F*	1.62		37.60		2.79		1.02		3.01	
	*p*	0.19		<0.01^**^		0.10		0.31		0.03^*^	
Weariness	*F*	6.89		95.46		8.52		0.04		1.78	
	*p*	<0.01^**^		<0.01^**^		< 0.01^**^		0.83		0.15	
Nausea and vomiting	*F*	1.41		85.69		0.00		0.22		2.47	
	*p*	0.24		<0.01^**^		0.98		0.64		0.06	
Pain	*F*	2.04		64.24		0.08		5.63		2.78	
	*p*	0.11		<0.01^**^		0.78		0.02^*^		0.04^*^	
General health	*F*	0.33		115.25		2.51		0.00		1.36	
	*p*	0.80		<0.01^**^		0.16		0.98		0.26	
Polypnea	*F*	3.32		40.20		0.29		1.65		0.98	
	*p*	0.02^*^		<0.01^**^		0.59		0.20		0.41	
Insomnia	*F*	1.67		38.31		0.26		0.10		3.68	
	*p*	0.18		<0.01^**^		0.61		0.76		<0.01^**^	
Inappetence	*F*	1.20		37.75		0.03		2.11		4.45	
	*p*	0.31		<0.01^**^		0.86		0.15		<0.01^**^	
Constipation	*F*	0.78		27.63		0.85		1.69		4.60	
	*p*	0.50		<0.01**		0.36		0.19		<0.01^**^	
Diarrhea	*F*	3.90		7.23		0.29		0.58		0.06	
	*p*	0.01^**^		0.01^**^		0.59		0.45		0.98	
Economic difficulty	*F*	0.75		9.44		0.66		0.09		1.02	
	*p*	0.52		<0.01^**^		0.42		0.76		0.38	

^a^Degree of freedom of numerator; ^b^Degree of freedom of denominator. ^*^p ≤ 0.05; ^**^p < 0.01.

**Table 4:** Fixed effects of quality of life results (*n* = 165).

	Group 1^a^			Group 2^b^			Group 3^c^			Time 1^d^			Group 1*time 1			Group 2*time 1			Group 3*time 1		
	*E* ^e^	*t*	*p* ^f^	*E*	*t*	*p*	*E*	*t*	*p*	*E*	*t*	*p*	*E*	*t*	*p*	*E*	*t*	*p*	*E*	*t*	*p*
Total score	−0.10	−3.40	<0.01^**^	−0.11	−3.81	<0.01^**^	−0.15	−5.16	<0.01^**^	0.02	1.08	0.28	0.10	3.49	<0.01^**^	0.08	2.89	<0.01^**^	0.12	4.20	<0.01^**^
Physical function	−0.09	−1.51	0.13	0.05	0.88	0.38	−0.11	−1.80	0.07	0.13	3.61	<0.01^**^	0.14	2.91	<0.01^**^	0.01	0.23	0.82	0.12	2.47	0.01^**^
Role function	−0.28	−3.27	<0.01^**^	−0.36	−4.42	<0.01^**^	−0.48	−5.83	<0.01^**^	−0.06	−1.00	0.32	0.40	4.91	<0.01^**^	0.36	4.34	<0.01^**^	0.56	6.81	<0.01^**^
Emotional function	−0.20	−3.08	<0.01^**^	−0.26	−4.20	<0.01^**^	−0.36	−5.73	<0.01^**^	−2 E-16	−0.00	1.00	2.56 E-16	0.00	1.00	3.32 E-16	0.00	1.00	2.02 E-16	0.00	1.00
Cognitive function	−0.28	−3.83	<0.01^**^	−0.40	−5.73	<0.01^**^	−0.29	−4.00	<0.01^**^	−0.12	−2.28	0.02^*^	0.24	3.33	0.00^**^	0.29	4.10	<0.01^**^	0.35	4.83	<0.01^**^
Social function	0.09	1.08	0.28	−0.11	−1.32	0.19	−0.14	−1.79	0.08	0.08	1.35	0.18	0.03	0.35	0.73	0.15	1.84	0.07	0.21	2.58	0.01^**^
Weariness	−0.18	−2.61	0.01^**^	−0.15	−2.32	0.02^*^	−0.29	−4.44	<0.01^**^	0.15	3.00	<0.01^**^	0.11	1.68	0.10	0.15	2.23	0.03*	0.09	1.29	0.20
Nausea and vomiting	−0.14	−1.73	0.09	−0.18	−2.26	0.03^*^	−0.16	−2.00	0.05^*^	0.14	2.60	0.01^**^	0.19	2.48	0.01^**^	0.08	1.05	0.30	0.16	2.08	0.04^*^
Pain	−0.25	−3.34	<0.01^**^	−0.12	−1.69	0.09	−0.19	−2.66	0.01^**^	0.09	1.71	0.09	0.19	2.52	0.01^**^	0.11	1.41	0.16	0.19	2.46	0.01^**^
Polypnea	−0.17	−2.03	0.04^*^	−0.05	−0.59	0.56	−0.26	−3.13	<0.01^**^	0.14	2.24	0.03^*^	0.04	0.51	0.61	0.03	0.37	0.71	0.14	1.62	0.11
Insomnia	−0.32	−3.28	<0.01^**^	−0.22	−2.34	0.02^*^	−0.21	−2.13	0.03^*^	0.01	0.22	0.82	0.27	2.86	<0.01^**^	0.23	2.43	0.02^*^	0.26	2.80	0.01^*^
Inappetence	−0.28	−3.01	<0.01^**^	−0.14	−1.53	0.13	−0.20	−2.12	0.04^*^	0.03	0.36	0.72	0.37	3.52	<0.01^**^	0.17	1.59	0.11	0.17	1.59	0.11
Constipation	−0.16	−1.36	0.18	−0.14	−1.59	0.11	−0.28	−3.10	<0.01^**^	0.03	0.51	0.61	0.12	1.30	0.20	0.10	1.03	0.31	0.17	1.59	0.11
Diarrhea	0.06	0.64	0.52	−0.16	−1.80	0.07	−0.04	−0.47	0.64	0.13	1.60	0.11	−0.02	−0.15	0.88	−0.03	−0.24	0.81	−0.05	−0.41	0.68
Economic difficulty	0.11	1.53	0.13	0.02	0.25	0.81	−0.01	−0.07	0.94	−0.01	−0.22	0.82	−0.13	−1.73	0.09	−0.06	−0.87	0.38	−0.08	−1.08	0.28
General health	0.09	1.31	0.19	0.07	1.07	0.29	0.12	1.81	0.07	−0.21	−3.64	<0.01^**^	−0.14	−1.80	0.07	−0.10	−1.20	0.23	−0.14	−1.71	0.09

^a^PMR group; ^b^Cognitive intervention group; ^c^Cognitive intervention plus PMR group; ^d^After the intervention; ^e^E = Estimate; ^f^Pr > |t|; ^*^p ≤ 0.05; ^**^p < 0.01.

For the 15 dimension scores, the interactions between intervention and time groups were observed (*p* < 0.05), except for emotional function, weariness, polypnea, inappetence, constipation, diarrhea, economic difficulty, and general health. As a covariate, ethnic group differences were insignificant for any dimension, except weariness (*p* > 0.05). Differences in disease course were insignificant for any dimension, except emotional function and pain (*p* > 0.05; Tables 3, 4).

In sum, cognitive intervention plus PMR significantly improved the overall QoL, four functional dimensions, two symptom dimensions, and insomnia.

### Changes in remission degree over time

The GLMM was built by setting remission degree as the response variable. Intervention group, time, ethnic group, disease course, and interaction between intervention and time were fixed effects, whereas individual factors were random effects. A significant difference was found in time, with different effects at different times (Type III fixed effects of remission over time: *F* = 13.49, *p* < 0.05). The interaction effects of the interventions were insignificant between intervention and time groups (*p* > 0.05; Tables 5).

**Table 5:** Fixed effects of remission results (*n* = 165).

Effect	Group	Time	Estimate	*t*	*p*
Intercept1			−2.66	−3.98	<0.01^**^
Intercept2			1.99	2.99	<0.01^**^
Group	1		−0.95	−1.22	0.22
Group	2		−0.65	−0.88	0.38
Group	3		−1.08	−1.43	0.16
Group	4		0		
Time		1	−1.15	−2.18	0.03^*^
Time		2	0		
Ethnic group			0.60	2.05	0.04^*^
Disease course			0.35	1.36	0.18
Group*time	1	1	0.18	0.24	0.81
Group*time	1	2	0		
Group*time	2	1	0.04	0.05	0.96
Group*time	2	2	0		
Group*time	3	1	0.46	0.61	0.54
Group*time	3	2	0		
Group*time	4	1	0		
Group*time	4	2	0		

Group 1: PMR group; Group 2: Cognitive intervention group; Group 3: Cognitive intervention plus PMR group; Group 4: Control group; Time 1: Immediately post-intervention; T2: Baseline. ^*^p ≤ 0.05; ^**^p < 0.01.

### Cost-effectiveness of the three psychological interventions

According to ICER calculation, considering that the estimated values for the PMR group, cognitive intervention group, and cognitive intervention plus PMR group were − 0.10, −0.11, and − 0.15, respectively, the costs of the three interventions were 1,850 RMB, 3,150 RMB, and 5,000 RMB. Thus, compared with cognitive intervention, the ICER value of cognitive intervention plus PMR was 46,250 RMB. Compared with the PMR, the ICER value of cognitive intervention plus PMR was 63,000 RMB. Compared with the PMR, the ICER value of cognitive intervention was 130,000 RMB. As no research and recommendation on willingness to pay (WTP) in China is currently found, our study adopted three times Chinese *per capita* in 2019 (70,892 RMB) according to WHO regulations, which is 212,676 RMB as WTP. Thus, ICER values indicated the cost-effectiveness of all three psychological interventions, among which the cognitive intervention plus PMR program showed the most significant cost-effectiveness advantage.

## Discussion

To our knowledge, our study is one of the few to conduct a psychological intervention combining cognitive intervention and PMR and examine its effects on QoL and remission in AL patients receiving chemotherapy. Our findings indicate that the QoL in AL patients receiving chemotherapy in the cognitive intervention plus PMR group was significantly improved compared with other groups. Cognitive intervention plus PMR is highly cost-effective for AL patients receiving chemotherapy in China.

### Effects of different interventions on the QoL of patients with AL treated with chemotherapy

The results showed that the cognitive intervention plus PMR group significantly improved the total QoL score and symptom dimensions scores, i.e., physical function, role function, and cognitive function, consistent with Persson et al. (2001). The reason may be through cognitive intervention, participants may have had strategies for handling various situations. As interventions were conducted at the initiation of chemotherapy, PMR–as a necessary relaxation therapy–in the combined intervention may prevent or considerably delay the onset of conditioned responses, therefore relieving distress to some degree (Tian et al., 2020). However, effects on emotional functions differ from Alibhai et al. (2012). This result may be because medical expenses are a considerable burden for most patients and their families, especially for patients in rural areas in China, weakening optimism and satisfaction with life. Psychological coping resources may also be poor without adequate financial resources (Zhang et al., 2009). Weariness insignificantly improves in intervention groups because it is a prominent symptom of AL survivors. After all, it interferes with daily living and the ability to perform social roles (Zhang et al., 2009). The other possible reason may be due to personality factors, supported by a previous systematic review that coping strategies depend on individual internal adaptation, on an unconscious level, resulting in life satisfaction, in combination with external adjustment, including actions on a conscious level, resulting in well-being (Leamy et al., 2011). Participants accustomed to a strong sense of experiential avoidance and self-defense may have difficulty generating timely internal adaptations and external actions, thus maintaining fatigue-related and emotional problems. Nevertheless, weariness improved over time from the start of treatment to the end of the study. In future studies, adding follow-up evaluations to further confirm these intervention effects in AL patients treated with chemotherapy is essential.

Based on these results, the cognitive intervention plus PMR group indicated the best outcomes with improvements in total QoL score, most dimensions, and the greatest cost-effectiveness. These results are consistent with Espie et al. (2008) and Hopko et al. (2008) and suggest that combined behavioral and cognitive interventions can tremendously improve well-being. The possible reason for the effects is the brief and group-based intervention. The brief cognitive intervention plus PMR intervention may make them less difficult to incorporate into existing medical systems and promote adherence. The group format may provide opportunities for social interaction and support along the leukemia trajectory, thus remarkably improving social function (Hunter et al., 2009). The other probable reason is that according to the stress theory of Lazarus and Folkman (1984), the cognitive evaluation of a stressful situation determines the coping behavior and affect what follows. Adding the PMR part further promotes participants’ ability to manage the difficulties they may face in the future. The effects of combined cognitive and muscle relaxation can also be supported by previous studies on different cancer patients (O’Dowd et al., 2006; Hunter et al., 2009).

Moreover, this study indicated that cognitive intervention improves the total QoL score, role and cognitive function, weariness and insomnia compared with the control group. Chemotherapy-associated cognitive dysfunction is an essential factor affecting prognosis, severely impairing QoL (Vetto and Vetto, 2007), with an overall consensus that cognitive therapy improves cancer patients’ QoL (Foley et al., 2010). AL and its treatment are adverse severe life events, which induce various negative emotions, impairing mental and physical health. Changing the cognitive process and concepts that emerge in this process, cognitive therapy corrects patients’ maladaptive feelings and behaviors, thus improving mental and physical health and treatment efficacy.

The PMR’s capacity to improve cancer patients’ QoL receiving chemotherapy was established in a previous study (Kwekkeboom et al., 2008). However, PMR had a weaker effect than cognitive intervention or cognitive intervention plus PMR in our study. Different from solely conducting PMR, adding a cognitive portion may help participants adjust to their internal experience, increase their endurance to the disease, and enhance self-efficacy through peer support. In turn, it promotes participants’ greater motivations to participate in interventions and make behavioral changes, finally improving their QoL.

Different intervention methods suggest that the psychological cognition of patients with AL significantly benefits their QoL more than PMR. A significant positive correlation exists between positive psychology and QoL in AL patients. Compared with PMR, which indirectly changes participants’ negative emotions through physical relaxation, cognitive therapy is nurses’ direct intervention in participants’ cognitive and psychological states that is more beneficial in relieving their negative emotions. The intervention combined with both methods further extends the intervention effects in this population.

### Effects of different interventions on the remission of patients with AL treated with chemotherapy

Cancer remission is of utmost importance to cancer patients and their families. Data show no significant difference among the four groups but a significant difference over time. Hence, the remission of patients with AL receiving chemotherapy is only marginally related to three interventions. Remission is closely associated with natural disease course and its treatment. These interventions do not change these patients’ remission degrees and survival rates. However, psychological interventions can improve QoL and patients’ general mental and physical conditions, permitting comfortable and dignified lives. Considering that QoL is closely related to disease remission rates and survival outcomes, different intervention effects on remission rates should be further investigated by increasing follow-up time points.

### Limitations

Several limitations should be noted in our study. First, the convenience sampling method and practical reasons may reduce the representativeness of the sample and the generalization of our findings. Second, the effects are only examined immediately after interventions due to time constraints and data collection challenges (e.g., missing participant contacts). Considering that the remission rate improvement cannot be instantaneous and cognitive intervention effects on QoL can strengthen over time as patients incorporate psychological intervention skills into everyday lives, long intervention procedures with multiple follow-up time points are recommended to confirm the effects and explore its maintenance effects in this population. Third, the intention-to-treat analysis was not adopted in this study; alternatively, per-protocol (PP) analysis was used to evaluate the effects of actually receiving interventions, which may not preserve the randomization and instead create bias. Thus, future studies may consider using intention-to-treat and PP analyzes and reporting alongside each other for comparison.

### Research and clinical practice implications

Our results provide implications for further research. First, cognitive intervention plus PMR is promising and cost-effective in improving QoL. More rigorous RCTs with follow-up time points are suggested to further clarify its effects in strengthening remission rates in AL patients treated with chemotherapy. Second, understanding patient experiences and perceptions during interventions may help refine existing intervention protocols. Therefore, qualitative studies are necessary to enrich our understanding of psychological intervention effects on this population. In addition, exploring the mechanisms of different psychological interventions in improving the QoL of AL patients receiving chemotherapy is profound to help clarify mediating intervention factors.

Our findings also provide several implications for clinical nursing practice. AL patients receiving chemotherapy experience severe physical, mental, and social strains imposed by the disease and treatment and decreased QoL, threatening their remission rates. Psychological interventions are highly recommended, especially cognitive–behavioral interventions. Our study confirms clinical nurses’ great potential in providing psychological support to oncology patients. In this case, clinical nursing administrators should pay further attention to training psychological nurses in oncology departments, making psychological support become a part of daily cost-effective routine care during hospitalization and after discharge. In addition, our study gives the initial support for providing AL patients with brief and group-based cognitive intervention plus PMR to improve their QoL. Hence, repeated bolus maintenance sessions (Trask et al., 2003) provided on a regular nursing basis may be beneficial in extending the initial change observed in the current study and tracking their progress.

## Conclusion

The cognitive intervention plus PMR intervention produces the best results for AL patients receiving chemotherapy with good cost-effectiveness. Cognitive intervention is an economical and convenient method when the required conditions are in place. The effect of PMR is the poorest among the three interventions, yet it is effective in improving QoL and can be used under certain circumstances. More rigorous RCTs with larger samples and longer follow-ups are recommended to further confirm these psychological intervention effects.

## Data availability statement

The original contributions presented in the study are included in the article/Supplementary material, further inquiries can be directed to the corresponding author.

## Ethics statement

The studies involving human participants were reviewed and approved by the study was reviewed and approved by the Xiangya School of Nursing, Central South University. The patients/participants provided their written informed consent to participate in this study.

## Author contributions

FP, HL, and JZ conceived the study. FP and XL collected and analyzed the data. JZ supervised the study conduction. FP and HL wrote the first draft of the manuscript. HZ and YL interpreted the results. All authors contributed to the article and approved the submitted version.

## Funding

The study was supported by Hunan Provincial Health Department (Project reference: B2007001) and Hunan Provincial Development and Reform Commission project (Project reference: 2007896).

## Conflict of interest

The authors declare that the research was conducted in the absence of any commercial or financial relationships that could be construed as a potential conflict of interest.

The handling editor FH declared a past co-authorship with the author JZ.

## Publisher’s note

All claims expressed in this article are solely those of the authors and do not necessarily represent those of their affiliated organizations, or those of the publisher, the editors and the reviewers. Any product that may be evaluated in this article, or claim that may be made by its manufacturer, is not guaranteed or endorsed by the publisher.
